# Pairing off: a bottom‐up approach to the human gut microbiome

**DOI:** 10.15252/msb.20188425

**Published:** 2018-06-21

**Authors:** Clare Abreu, Anthony Ortiz Lopez, Jeff Gore

**Affiliations:** ^1^ Department of Physics Massachusetts Institute of Technology Cambridge MA USA; ^2^ Microbiology Graduate Program Massachusetts Institute of Technology Cambridge MA USA

**Keywords:** Microbiology, Virology & Host Pathogen Interaction, Quantitative Biology & Dynamical Systems, Synthetic Biology & Biotechnology

## Abstract

The human gut microbiome has been implicated in a variety of health outcomes, and extensive research has aimed to understand its composition and function, primarily via metagenomic analyses. An examination of how the microbiome develops and interacts through interspecies competition and cooperation has been lacking so far. In their recent work, Venturelli *et al* (2018) build a synthetic gut community and accurately predict its dynamics with a simple network of pairwise interactions.

Imagine if a single organ of the human body was proposed to be implicated in a wide variety of diseases and disorders, including Parkinson's disease, autism, depression, obesity, ulcerative colitis, cardiovascular disease, diabetes, and multiple cancer types. The gut microbiome, a microbial ecosystem inhabiting the digestive tract of humans and other animals, is frequently referred to as an additional organ since its weight (~1 kg) is comparable to that of a small organ, and its role in physiology and disease has become evident in the recent years. In fact, the gut microbiome has been linked to all the conditions listed above as well as further pathologies (Shreiner *et al*, 2015). While several of the proposed links between the microbiome and human diseases remain to be further investigated and might after all prove to be overblown, they nevertheless have drawn tremendous attention to the gut microbiome, and research continues at a fever pitch to link the microbiome to health outcomes. Metagenomic analyses have facilitated a “top‐down” look at gut community composition (Human Microbiome Project Consortium, 2012), but “bottom‐up” approaches can be instrumental for understanding how these complex bacterial communities form, interact, and affect the health of their host. In their recent study, Venturelli *et al* (2018) use a bottom‐up approach to community assembly by performing experiments with a synthetic gut microbial community and analyze the data using a predictive dynamic computational model. They find that pairwise interactions are sufficient to model multispecies community assembly and that certain pairwise motifs may be key to making a healthy microbiome resilient over time.

Although the authors’ model community is less complex and diverse than real gut microbiome communities, its 12 species span the major phyla of human‐associated intestinal bacteria. Venturelli *et al* (2018) performed all possible two‐species combination experiments and tracked the relative abundances across time with multiplexed 16S sequencing and the total biomass with OD600 measurements. By fitting the pairwise dynamics to a generalized Lotka–Volterra model, they estimated the network of interactions (Fig 1) and were able to accurately predict the majority of temporal changes in 11‐species (single‐species dropout communities) and 12‐species (full communities) communities. The predictions from pairwise experiments were significantly better compared to predictions based on single‐species growth. This finding agrees with work from our group (Friedman *et al*, 2017) and others (Guo & Boedicker, 2016) showing that pairwise outcomes are adequate for predicting multispecies community assembly and community‐level metabolic rates. In contrast to some theoretical work and empirical evidence (Bairey *et al*, 2016; Grilli *et al*, 2017; Mayfield & Stouffer, 2017), these results suggest that higher‐order interactions may play a relatively minor role in driving community assembly and that a network of pairwise interactions is an adequate description of the system.

**Figure 1:**
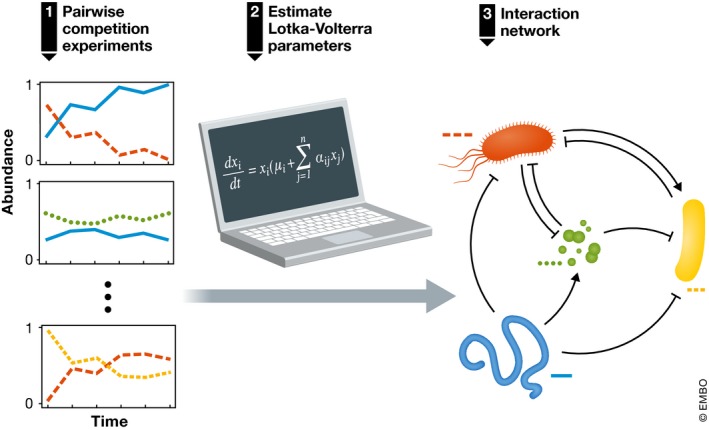
Interaction network of microbiome constructed from pairwise dynamics The temporal dynamics of two‐species bacterial communities were used by Venturelli *et al* (2018) to estimate the parameters of a Lotka–Volterra model. The parameters of this model represent growth rates, intra‐ and interspecies interactions, and the interspecies interaction coefficients can be used to visualize the microbial ecological network.

The types of links that connect pairs of bacteria in the network influence the ecological dynamics of the communities in which they are embedded. The authors found that approximately half of the interactions are negative (56 vs. 21% positive) and note that, generally, negative interactions introduce stabilizing feedbacks to networks. Negative interactions are generally thought to arise from competition for resources or direct conflict involving toxin production, while positive interactions can occur when one species secretes a metabolite utilized by another species, a process known as “cross‐feeding.” However, even net‐negative interactions could contain some cross‐feeding, a concept similar to a casino offering free drinks to gamblers, making a negative interaction less negative. The prevalence of negative interactions in the network is in line with previous results from studies of culturable microbes (Foster & Bell, 2012).

Studying pairwise dynamics may be useful for predicting the survival of an invading pathogen or probiotic, or on the other hand, the effects of removing a species from a community. The authors found that several of the analyzed species, which happened to be the strongly interacting and abundant ones, had a dramatic effect on the structure of the 11‐ and 12‐species communities. These results suggest that pairwise interactions are important in understanding how a gut community would respond to a species invasion or removal. Unhealthy gut microbiota states have been shown to be less stable to perturbations (Sommer *et al*, 2017). The authors noted that the prevalence of negative interactions in the network could be a stabilizing force and, more specifically, they highlighted a particular pairwise motif that appeared frequently. The positive/negative interaction motif was associated with stable coexistence in experiments and simulations and is one potential mechanism that may lead to emergent stability of a community.

The advantage of analyzing synthetic communities is that they are experimentally tractable. However, the tradeoff of this controllability is the uncertainty of how well the findings apply to real, complex gut communities. Experiments performed in liquid cultures have less spatial structure than natural intestinal systems, and such spatial separation has been shown to promote coexistence. Additionally, serial transfer experiments use a dilution rate to model colonic transit time, but this approximation might err in its simplicity. Notwithstanding these differences, the relative abundance of species appearing in both experiments and real human guts was found to be correlated.

The work of Venturelli *et al* (2018) contributes to the ongoing effort to understand the ecology of the human microbiome. The insightful results from a synthetic model community highlight the merits of applying a “bottom‐up” approach to the analysis of the gut microbiome. Nontrivial patterns emerge from the 12‐species network, built by simple two‐species competition experiments. The use of a simple phenomenological model was also validated by its accurate predictions of community assembly and dynamics. While such models are considered to be context‐dependent in comparison with mechanistic models, which incorporate more information such as metabolomics data, in this case the authors reported that fewer predictive insights were gained from metabolite measurements. Future experiments may reveal why some communities are more easily perturbed than others and whether the “healthy state” of a microbiome community can be explained by its pairwise network. Such experiments could test the predictions of Venturelli and colleagues, bridging synthetic and natural communities.
